# Differentiation of primary central nervous system lymphoma from high-grade glioma and brain metastases using susceptibility-weighted imaging

**DOI:** 10.1002/brb3.288

**Published:** 2014-10-10

**Authors:** Yaling Ding, Zhen Xing, Biying Liu, Xinjian Lin, Dairong Cao

**Affiliations:** 1 Department of Radiology, First Affiliated Hospital, Fujian Medical UniversityFuzhou, 350005, China; 2 Department of Medicine and UC San Diego Moores Cancer Center, University of California-San DiegoLa Jolla, California, USA

**Keywords:** Brain metastases, differential diagnosis of intracranial malignancies, high-grade glioma, primary CNS lymphoma, susceptibility-weighted imaging

## Abstract

**Background and Purpose:**

Conventional MRI is often difficult to distinguish between primary central nervous system lymphomas (PCNSLs), high-grade gliomas and brain metastases due to the similarity of their appearance. The aim of this study was to investigate whether the susceptibility-weighted imaging (SWI) has higher sensitivity than conventional MRI in detecting hemorrhage between PCNSLs, high-grade gliomas and brain metastases, and can be used to differentiate the diagnosis between these tumors.

**Methods:**

The number of lesions with hemorrhage was quantified by both the conventional MR imaging and SWI. The number of micro-hemorrhage and vessels within lesions were counted on SWI.

**Results:**

The detection rate of hemorrhage on SWI was significantly higher than that on the conventional MR imaging. The intralesional hemorrhagic burden and the number of the vessels within lesions detected by SWI were significantly higher in high-grade gliomas and brain metastases than those in PCNSLs. There was no significant difference in these two parameters between high-grade gliomas and brain metastases. The best predictor to differentiate PCNSLs from high-grade gliomas and brain metastases was intralesional vessel number that yielded the best ROC characteristics and highest classification accuracy.

**Conclusions:**

SWI is useful in differentiating of PCNSLs from high-grade gliomas and brain metastases.

## Introduction

Primary central nervous system lymphomas (PCNSLs) account for 5% of all primary brain tumors (Tang et al. 2011). Although PCNSLs demonstrate some of characteristic magnetic resonance imaging (MRI) findings, their MR imaging features can vary with immune status and histological type and often overlap with other intracranial tumors making definitive diagnosis challenging (Liu et al. 2009; Tang et al. 2011). In particular, it is difficult or even impossible to distinguish between PCNSLs, high-grade gliomas and brain metastases due to the similarity of their appearance on the conventional MR imaging (Buhring et al. 2001; Coulon et al. 2002; Kuker et al. 2005).

However, an accurate diagnosis and differentiation is crucial since prognosis and therapy are completely different for PCNSLs, high-grade gliomas and brain metastases. Susceptibility-weighted imaging (SWI) is a new MR imaging technology developed in recent years. Differing from the conventional T1WI and T2WI, SWI is sensitive to T2* caused by local susceptibility effects (Sehgal et al. 2005). This new technology has been proved to be a very valuable tool in the assessment of intracranial mass lesions. SWI is able to demonstrate the differences of tissues with susceptibilities and provide excellent contrast between blood products, venous blood vessels, iron-laden and calcification distinguishable from the surrounding tissues, which is not provided by conventional MR imaging (Haacke et al. 2004; Sehgal et al. 2006; Xu and Haacke 2006; Radbruch et al. 2013).

In fact, hemorrhage and infarction was rarely seen in PCNSLs probably because of outstripping of its blood supply. It has been shown that cerebral lymphomas tended to have low relative cerebral blood volume (rCBV) values (Sugahara et al. 1999) and appeared to be significantly lower than those of enhancing high-grade glioblastomas and brain metastases (Cho et al. 2002). Similarly, the microvessel density, a pathological feature closely related to rCBV value, was found much lower in PCNSLs than in malignant gliomas (Liao et al. 2009). Therefore, in this study we use SWI to evaluate blood products and vasculature within brain tumors and assess whether SWI can be used to distinguish PCNSL, high-grade gliomas and brain metastases.

The purpose of this study is to evaluate the advantages of using SWI to detect and characterize PCNSLs, high-grade gliomas and brain metastases for differential diagnosis in comparison to conventional MR sequences. We hypothesized that the difference in the number of hemorrhage and vessels within lesions would allow differentiation between these three tumors.

## Methods

### Study subjects

A retrospective analysis of our database of 79 patients who had undergone SWI in addition to conventional MR imaging in September 2009–February 2012 was performed. In this study, the lesions with large sheets of hemorrhage were excluded because multiple micro-hemorrhage and vessels are not distinguishable from each other on SWI and could be mis-regarded as large sheets of hemorrhage. None of these patients had previously received radiotherapy, chemotherapy or surgical treatment for brain tumor. The Institutional Review Board of our hospital approved this retrospective study and patient informed consent was waived.

A total of 104 lesions with a mean diameter of 3.5 ± 1.6 cm ranging from 1.5 to 7.3 cm were enrolled in the study which included 23 lesions with PCNSLs (15 patients, four male, 11 female; mean age, 56.0 ± 13.0 years with a range from 29 to 72 years), 35 lesions with high-grade gliomas (30 patients, 18 male, 12 female; mean age, 50.2 ± 14.3 years with a range from 26 to 74 years) and 46 lesions with brain metastases (34 patients, 20 male, 14 female; mean age, 58.6 ± 11.5 years with a range from 24 to 78 years). All the tumors were confirmed by histopathological examination after surgical resection or biopsy and classified according to the new WHO Classification of Tumours of the Central Nervous System, Fourth Edition. Each of the 15 patients with PCNSLs had diffuse large B-cell lymphomas as confirmed by histopathological examination after biopsy but did not have immunodeficient diseases. Of the 35 lesions with high-grade gliomas, 11 lesions (31.4%) were grade III gliomas and 24 lesions (68.6%) were grade IV gliomas. 46 brain metastases from the known primary cancers were also biopsy-proved. Among the 46 biopsied cases, 32 originated from the lung, four from the breast, three from kidney, three from the stomach, two from cervix, one from liver and one from colon.

### Imaging protocol

All the MR images were acquired on a 3.0 T scanner (SIEMENS Verio 3.0T, Erlangen, Germany) with an 8-channel head coil. Conventional MR examinations included spin-echo T1-weighted imaging (TR/TE 250 ms/2.48 ms) and fast spin-echo (FSE) T2-weighted imaging (TR/TE 4000 ms/96 ms). The other MR sequences used included DWI (TR/ TE 8200 ms/102 ms, Average = 1, *b* value = 1000s/mm^2^), FLAIR (TR 9000 ms, TE 94 ms, TI 2500 ms) and contrast-enhanced T1-weight (gadopentetate dimeglumine: Magnevist; Schering, Berlin, Germany; injection: 0.1 mmol/kg). Imaging parameters of SWI were as follows: TR/TE 27/20 ms, flip angle 15°, field of view (FOV) 23 cm × 23 cm, section thickness 1.5 mm, matrix 256 × 256, reconstruction = Magn/phase. Susceptibility- weighed images were reconstructed by the corrected phase images and magnitude images. Adjacent magnitude images were post-processed into a minimum intensity projection (MinIP) setting with the slice thickness of 2 mm. SWI is performed in the same imaging session with T1WI and T2WI as the routine workup for tumors in the preoperative patients.

### Image analysis

All MR images were reviewed by two radiologists (D. C., Z. X., 15 and 5-year experience respectively) blinded of surgical or pathological results. The imaging features analysis included tumor location, numbers, signal, hemorrhage, and vessels within lesions. In SWI-MinIP imaging that covers whole tumor area, dot-like hypointense signal was regarded as microhemorrhage, while linear hypointense signal was regarded as intralesional vessels which would be followed on consecutive SWI images. The number of lesions with hemorrhage was counted on the conventional MR imaging and SWI. The number of intralesional hemorrhage and vessels was also counted and graded on SWI. The intralesional hemorrhagic burden was estimated on SWI as Grade 0: no hemorrhage, Grade 1: 1–10 dot-like hemorrhage, Grade 2: 11–20 dot-like hemorrhage, Grade 3: more than 20 dot-like hemorrhage. The intralesional vessel score on SWI was counted and divided into four grades: Grade 0: no intralesional vessels; Grade 1: 1–5 intralesional vessels; Grade 2: 6–10 intralesional vessels; Grade 3: more than 10 intralesional vessels.

### Statistical analysis

All the statistics were analyzed by SPSS software (SPSS 17.0 version for windows, SPSS Inc., Chicago, IL, USA). The interobserver variability in determining the value of hemorrhage and tumor vascularity by two physicians was assessed by intraclass correlation test (ICC). If the interobserver variability has an excellent agreement (*ICC>0.75)*, the views of this two physicians were intergraded through taking the average of data. High-grade gliomas and brain metastases were first grouped together as non-PCNSLs (vs. PCNSLs) and further subclassified into high-grade gliomas and brain metastases respectively. Differences in the number of lesions with hemorrhage between conventional MR imaging and SWI was analyzed by Chi-square test. On SWI, the comparison of the intralesional hemorrhagic burden and the numbers of the vessels within lesions between PCNSLs and non-PCNSLs or between high-grade gliomas and brain metastases was analyzed by Wilcoxon rank sum test. Receiver operating characteristic (ROC) was also performed to differentiate PCNSL from high-grade gliomas and brain metastases. We defined a diagnostic test (positive vs. negative) for each type of tumors using a cutoff threshold for each of SWI parameters. The optimal cutoff (Youden index) was selected to maximize the sum of the sensitivity and specificity. Areas under the ROC curves (AUCs) were estimated and compared by chi-square test and the values greater than 0.70 were considered relatively high accuracy. *P *<* *0.05 was considered statistically significant.

## Results

### Characteristic imaging features of PCNSL, high-grade gliomas and brain metastases on the conventional MR imaging and SWI

**Figure 1:**
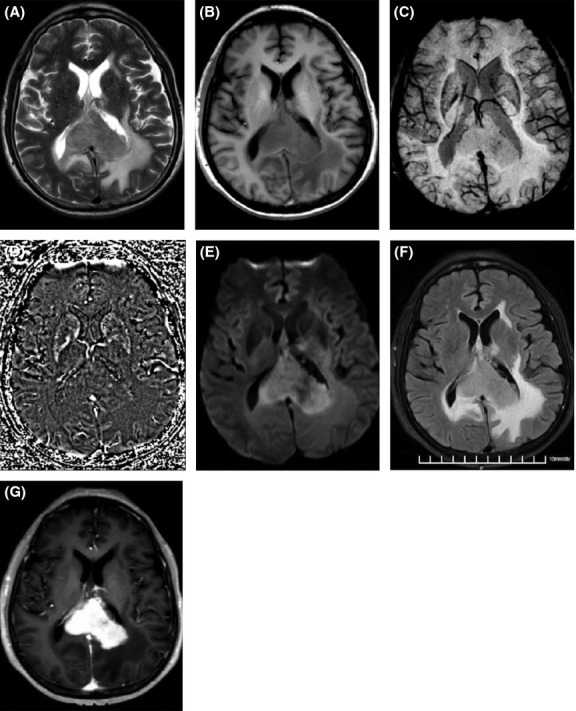
A 62-year-old male with diffuse large B cell lymphoma. One lesion was located in splenium on T2WI (A) and T1WI (B). There were no vessels and microhemorrhage shown on T1WI and T2WI. However, SWI-MinIP (C) showed microhemorrhage with sharp border in the lesion and high signal on phase image. (D) The lesion showed hyperintense signal both on DWI (E) and FLAIR (F), and demonstrated solid, intense, and homogeneous enhancement on contrast-enhanced T1-weighted image (G).

**Figure 2:**
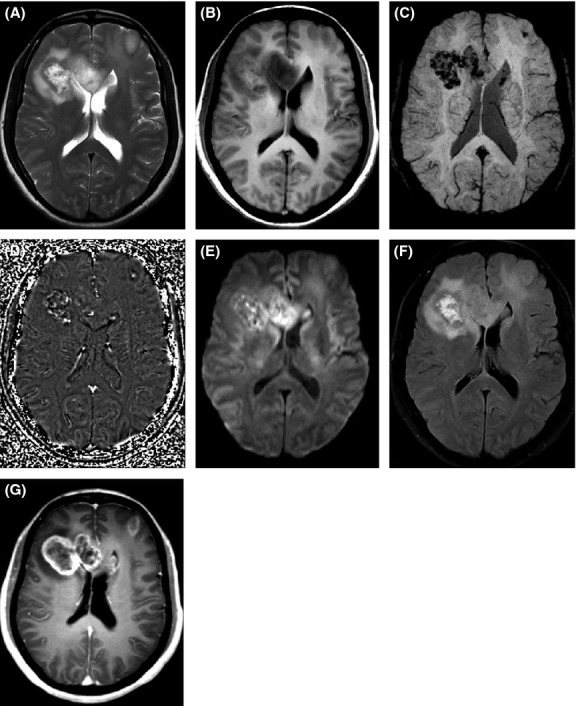
A 45-year-old female with glioblastoma (WHO grade IV). Three lesions were located in bilateral frontal lobe and genu corpus callosum on T2WI (A) and T1WI (B). In the lesion located in the right frontal lobe, the 2 dot-like low signals were found on T2WI. However, on SWI-MinIP (C), more hemorrhagic signals were found in lesions, which showed high signals on phase image (D), and a vessel was found in the lesion located in genu corpus callosum. The lesions showed hyperintense signal both on DWI (E) and FLAIR (F), and demonstrated ring, intense and inhomogeneous enhancement on contrast-enhanced T1-weighted image (G).

**Figure 3:**
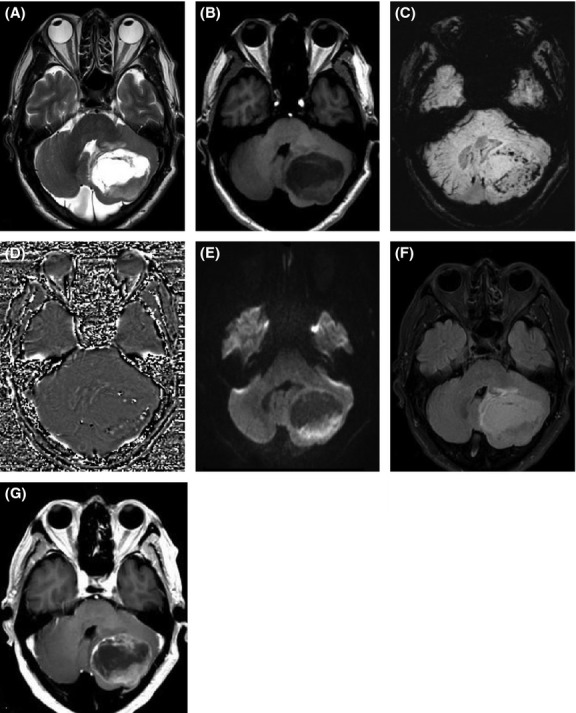
A 60-year-old female with metastatic neuroendocrine carcinoma (the primary sites of cancer was lung). One lesion was located in left cerebellar hemisphere on T2WI (A) and T1WI (B). There were no vessels and microhemorrhage shown on T1WI and T2WI. However, on SWI-MinIP (C), multiple hypointense signals regarded as hemorrhage and vessels were found in the lesion, which showed hyperintense signal on phase image (D). The lesion showed hyperintense signals both on DWI (E) and FLAIR (F), and demonstrated solid, intense and inhomogeneous enhancement on contrast-enhanced T1-weighted image (G).

The representative MR images of a patient with PCNSL, high-grade glioma or brain metastases were shown in Figs.1–3 respectively. On conventional imaging, PCNSLs, high-grade gliomas and brain metastases displayed similar imaging features as all the lesions were typically iso to hyperintense on T2WI but often hypointense on T1WI. On DWI, lesions with PCNSLs and high-grade gliomas showed different degree of hyperintense signal whereas brain metastases varied from low to high signal Intensity. On FLAIR, most of the malignant metastases presented as hyperintense masses surrounded by mild to massive vasogenic edema, and all the metastatic lesions showed ring or solid enhancement on contrast-enhanced T1-weighted images. On SWI, dot-like and linear hypointense signal was found in almost all the lesions with high-grade gliomas and brain metastases while only a few hypointense signals were seen in some lesions with PCNSLs.

### The interobserver variability in determining the value of hemorrhage and tumor vascularity by two physicians

To determine the interobserver MR variability, all the data were evaluated by two physicians. On the conventional MR imaging, the numbers of lesions with hemorrhage observed by physicians 1 and 2 were in complete agreement. And the same thing emerged on SWI (Table 1). Furthermore, the interobserver difference in determining the number of hemorrhage and intralesional vessels on SWI was assessed by intraclass correlation test. The mean number of hemorrhage was 7.83 ± 8.69 and 7.70 ± 8.58 for observers 1 and 2 respectively. The mean number of intralesional vessels was 3.76 ± 4.69 and 3.62 ± 4.35 for observers 1 and 2 respectively. According to the results of intraclass correlation test, the difference between observers was not great enough to exclude the possibility that it was due to chance (ICC* =* 0.998 and 0.991 for the difference in the number of hemorrhage and intralesional vessels respectively). Then the views of these two physicians were intergraded through taking the average of data and graded them according to the criterion as noted above (Tables 2–5).

**Table 1:** The detection rate of hemorrhage within lesions by the conventional MR imaging and SWI

Examination	Hemorrhage	No hemorrhage	Total	Detection rate
Conventional MR imaging	44	60	104	42.3%
SWI	79	25	104	76.0%
Total	123	85	208	59.1%

*χ*^2 ^= 24.37, *P < *0.005.

**Table 2:** The intralesional hemorrhagic burden in PCNSLs and non-PCNSLs on SWI

Grade of hemorrhage	PCNSLs	Non-PCNSLs	Total
0	16	10	26
1	7	42	49
2	0	19	19
3	0	10	10
Total	23	81	104

*u = *5.319, *P < *0.0005.

**Table 3:** The number of intralesional vessels in PCNSLs and non-PCNSLs on SWI

Grade of Intralesional Vessels	PCNSLs	Non-PCNSLs	Total
0	23	14	37
1	0	39	39
2	0	19	19
3	0	9	9
Total	23	81	104

*u = *13.83*, P < *0.0005.

**Table 4:** The grade of hemorrhage in high-grade gliomas and brain metastases on SWI

Grade of hemorrhage	High-grade gliomas	Brain metastases	Total
0	5	4	9
1	16	27	43
2	8	11	19
3	6	4	10
Total	35	46	81

*u = *0.1747*, P > *0.05.

**Table 5:** The number of intralesional vessels in high-grade gliomas and brain metastases on SWI

Grade of intralesional vessels	High-grade gliomas	Brain metastases	Total
0	6	8	14
1	14	25	39
2	8	11	19
3	7	2	9
Total	35	46	81

*u = *1.319*, P > *0.05.

### Comparison of the detection rate of hemorrhage within lesions between the conventional MR imaging and SWI

In 104 lesions enrolled, hemorrhage was found in 44 (42.3%) lesions on the conventional MR imaging and 79 (76.0%) lesions on SWI. Chi-square test showed that there was a statistically significant difference in the detection rate of hemorrhage between the conventional MR imaging and SWI (*P < *0.005*,* Table 1).

### Comparison of the intralesional hemorrhagic burden and the number of intralesional vessels between PCNSLs and non-PCNSLs on SWI

**Figure 4:**
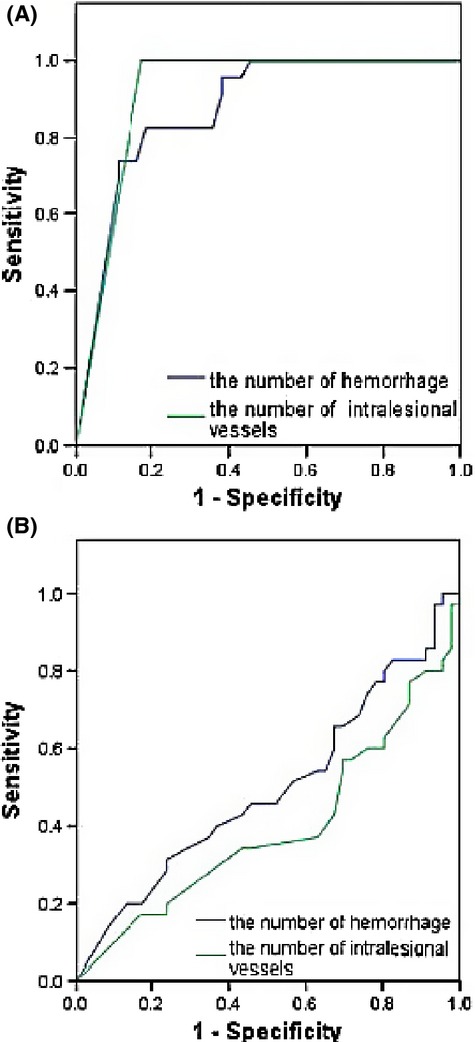
(A) Receiver operating characteristic for comparison of the number of hemorrhage and intralesional vessels for differentiation of PCNSLs from non-PCNSLs. The AUC for the number of hemorrhage and intralesional vessels is 0.873 (95% CI, 0.802–0.945) and 0.914 (95% CI, 0.860–0.967) respectively. (B) ROC for comparison of the number of hemorrhage and intralesional vessels for differentiation of high-grade gliomas and brain metastases. The AUC for the number of hemorrhage and intralesional vessels is 0.495 (95% CI, 0.365–0.625) and 0.393 (95% CI, 0.265–0.521) respectively.

Tables 2 and 3 summarized the results of grading the intralesional hemorrhagic burden and the number of intralesional vessels in the lesions with PCNSLs and non-PCNSLs detected by SWI. Wilcoxon rank sum test showed that the intralesional hemorrhagic burden (*P *<* *0.0005, Table 2) and the number of intralesional vessels (*P *<* *0.0005, Table 3) in non-PCNSLs was statistically higher than PCNSLs. ROC curve analysis for the number of hemorrhage in the differentiation of PCNSLs from non-PCNSLs demonstrated that cutoff value, optimal sensitivity, specificity, positive predictive value (PPV), and negative predictive value (NPV) was 2.5, 82.6%, 81.5%, 55.9%, and 94.3% respectively. The number of intralesional vessels was also very helpful in the differentiation of PCNSLs from non-PCNSLs with an optimum cutoff of 0.5, providing the sensitivity, specificity, PPV, and NPV of 100.0%, 82.7%, 62.2%, and 100.0% respectively (Fig.4A). The number of intralesional vessels appeared to be a better criterion than the number of hemorrhage in distinguishing PCNSLs from high-grade gliomas and brain metastases.

### Comparison of the intralesional hemorrhagic burden and the number of intralesional vessels between high-grade gliomas and brain metastases on SWI

In the lesions with high-grade gliomas and brain metastases, the results of grading the intralesional hemorrhagic burden and the number of intralesional vessels by SWI were shown in Tables 4 and 5. There was no statistically significant difference in these two parameters between these two tumors (*P *>* *0.05). From the ROC analysis a threshold value of 3.75 for the number of hemorrhage optimized differentiation of high-grade gliomas and brain metastases with a sensitivity, specificity, PPV, and NPV of 31.4%, 76.1%, 50.0%, and 59.3% respectively (Fig.4B). A threshold value of 0.50 for the number of intralesional vessels provided sensitivity, specificity, PPV, and NPV of 17.1%, 82.6%, 42.9%, and 56.7% respectively (Fig.4B). Thus, these two parameters may not be useful in the differentiation between high-grade gliomas and brain metastases (AUC < 0.5).

## Discussion

PCNSL has been documented to possess many imaging features which may mimic other diseases or have atypical imaging characteristics. In a recent study of 26 PCNSL patients, only 39% of cases were accurately diagnosed on MR imaging alone (Zhang et al. 2010). In addition, owing to their diffuse infiltrative or invasive growth, PCNSL, high-grade glioma and brain metastases display similar MR patterns. Therefore, it is often difficult, sometimes even impractical, to differentiate these three tumors by the conventional MR imaging. Our study illustrates the utility of different hemorrhage extent and vascularity in a SWI measurement for differentiating the three common enhancing malignant lesions of the brain. The results of this study demonstrate the high hemorrhage extent and intralesional vessel number in high-grade gliomas and metastases but low in PCNSLs, suggesting that these two parameters are effective in eliciting distinctive characteristics for the differentiation and may aid in the clinical management of these malignant tumors of the brain.

SWI technique is a novel imaging method that maximizes the sensitivity to susceptibility effects by combining a long-TE, high-resolution, fully flow-compensated, 3D gradient-echo sequence with filtered phase information in each voxel to both enhance the contrast in magnitude images and add a new source of information: the susceptibility difference between tissues (Reichenbach et al. 1997; Sehgal et al. 2006). SWI utilizes paramagnetic blood deposition as an intrinsic contrast agent which causes a reduction in T2* as well as a phase difference between the vessels and their surrounding brain tissues (Reichenbach et al. 1997). Microhemorrhages contain various kinds of blood products which have a remarkable hypointense signal and diminished T2* on SWI (Gomori et al. 1987). These characteristics render SWI exquisitely sensitive to detect the venous vasculature (Reichenbach et al. 2000) blood products and vascular malformations (Lee et al. 1999; Essig et al. 2001). While traditional T1 postcontrast imaging only could detect tumor necrosis, cystic changes and the extent of blood-brain barrier breakdown it cannot tell the details of intratumoral architecture and neovascularity due to the hyperintensity of contrast medium. FLAIR imaging can depict a large fraction of the tumor but lack specificity (Al-Okaili et al. 2007; Cha 2009). In addition, SWI is more sensitive and specific than T2*-gradient-recalled echo (GRE) because it has less tendency for artifacts (Lobel et al. 2010) allowing a more precise demonstration of the size and number of lesions. SWI employs both magnitude and filtered-phase information to generate an image based on T2* contrast and filtered phase changes caused by magnetic susceptibility. When the SWI images forms from the phase images multiplied with the magnitude images, the hemorrhage and venous data is enhanced (Sehgal et al. 2005). Then the SWI images are processed into SWI-MinIP images. Our data were collected at 3.0 T MR imaging equipment such that the high-field (3.0 T) SWI allows for optimal susceptibility effects at shorter measurement times, having advantages for obtaining detailed and high spatial resolution images of blood products and vessels network. Therefore, SWI-MinIP showed a clear improvement at imaging small vessels and hemorrhage over the conventional MR imaging in our study.

Vascular proliferation is one of the most essential factors in the biological behaviors of malignant brain tumors (Chaudhry et al. 2001). It is generally accepted that the more malignant the glioma, the greater the degree of hemorrhage and intralesional vasculature (Scatliff et al. 1969, 1971). It has also been reported that brain metastases increased the tumor microvascularity and neovascularity leading to increased relative cerebral blood volume (rCBV) during the process of growth and invasion (Cho et al. 2002; Hakyemez et al. 2006). In contrast with the high-grade gliomas and brain metastases, PCNSL is scarce in tumor neovascularization. On conventional angiography, PCNSL is usually present as an avascular mass, which is classically considered to be helpful for distinguishing itself from other malignant tumors abundant in neovasculatures (Helle et al. 1984; Frank et al. 1985). Therefore, differences in vascular proliferation between PCNSL and high-grade gliomas or brain metastases provide the basis for possible differential diagnosis made by SWI.

The architecture within tumor is discernible on SWI-MinIP, pointing toward the importance in guiding biopsy position before operating to avoid a qualitative error. On SWI-MinIP, we found hemorrhage and vessels all present as low-signal regions but high-signal regions on the filtered phase images. However, microhemorrhage was dot-like in shape whereas the vessel appeared linear that can be followed on contiguous slices. This discrepancy in their appearance may provide additional information for the discrimination. In line with other studies (Sehgal et al. 2006; Liao et al. 2009; Hori et al. 2010) indicating that SWI is more sensitive to detect hemorrhage than the conventional MR imaging, we found hemorrhage in 76.0% lesions on SWI but only in 42.3% of the same lesions on the conventional MR imaging, reflecting nearly twofold increase in the sensitivity by SWI. Our results clearly show that SWI is better than the conventional MR imaging in terms of detecting hemorrhage, and as expected, PCNSLs appeared to have dramatically lower incidence of hemorrhage and number of vessels compared with high-grade gliomas and brain metastases, as measured by SWI. This result is in good agreement with those of early reports (Helle et al. 1984; Frank et al. 1985; Liao et al. 2009) and support the notion that the absence of neovascularity in PCNSLs could be utilized to differentiate from high-grade gliomas or brain metastases. However, there are no significant differences in intralesional vascularity between high-grade gliomas and brain metastases. ROC, which evaluated the diagnostic performance of hemorrhage extent and intralesional vessel number, showed that the single best predictor for differentiating PCNSLs from non-PCNSLs was the number of intralesional vessels with AUC = 0.914, sensitivity = 1.00, and specificity = 0.83. In contrast, neither the hemorrhage extent nor intralesional vessel number was a good parameter in differentiating high-grade gliomas from brain metastases in the ROC curve analyses (AUC < 0.5, *P *>* *0.05). It should also be recognized that the nature of primary tumors would probably affect the vascularity of their metastases in brain and therefore other types of the metastatic tumors than those included in this study may lead to a different result.

Although cautions should be exercised against overinterpretation of the imaging parameters obtained from this study and against applying these parameters to the differentiation between high-grade gliomas and brain metastases, our results suggest that quantitative analysis of intratumoral hemorrhage and vessels from SWI imaging adds specificity to noninvasive differentiation of PCNSLs from high-grade gliomas and brain metastases, which may have considerable overlap in their conventional MR imaging appearance.
